# Effects of Fumonisin-Contaminated Corn on Growth Performance of 9 to 28 kg Nursery Pigs

**DOI:** 10.3390/toxins12090604

**Published:** 2020-09-18

**Authors:** Zhong-Xing Rao, Mike D. Tokach, Jason C. Woodworth, Joel M. DeRouchey, Robert D. Goodband, Hilda I. Calderón, Steve S. Dritz

**Affiliations:** 1 Department of Animal Sciences and Industry, College of Agriculture, Kansas State University, Manhattan, KS 66506, USA; zxrao@ksu.edu (Z.-X.R.); Mtokach@ksu.edu (M.D.T.); jwoodworth@ksu.edu (J.C.W.); jderouch@ksu.edu (J.M.D.); 2 Department of Statistics, College of Arts and Sciences, Kansas State University, Manhattan, KS 66506, USA; hilda1@ksu.edu; 3 Department of Diagnostic Medicine/Pathobiology, College of Veterinary Medicine, Kansas State University, Manhattan, KS 66506, USA; dritz@vet.ksu.edu

**Keywords:** fumonisin, growth, mycotoxins, nursery pigs, sphinganine, sphingosine

## Abstract

Fumonisin contamination in corn is an emerging issue in animal feed production. Fumonisin disrupts the metabolism of sphingolipids and reduces growth performance. This experiment was conducted to determine the effect of feeding fumonisin-contaminated corn on growth performance and sphinganine (SA) to sphingosine (SO) ratios of 9 to 28 kg pigs. A total of 350 pigs, were used with 5 pigs/pen and 14 pens/treatment. Dietary treatments contained fumonisin-contaminated corn (50 mg/kg of fumonisin B1 + B2) blended with low fumonisin corn (10 mg/kg of fumonisin B1 + B2) to provide dietary fumonisin concentrations of 7.2, 14.7, 21.9, 32.7, and 35.1 mg/kg. From day 0 to 28, increasing fumonisin concentration decreased (linear, *p* < 0.001) average daily gain, average daily feed intake (linear, *p* = 0.055), and gain:feed ratio (linear, *p* = 0.016). Although these response criteria tested linear, the greatest reduction in performance was in pigs fed with 32.7 and 35.1 mg/kg of fumonisin (B1 + B2). Increasing fumonisin concentration increased the serum SA:SO ratio (linear, *p* < 0.001) on day 14 and 28. In summary, for 9 to 28 kg nursery pigs, increasing fumonisin linearly decreased average daily gain and gain:feed ratio. However, despite the linear response, diets containing up to 21.9 mg/kg of fumonisin did not have as dramatic a decrease in growth performance as those fed more than 32.7 mg/kg. Further research is warranted to determine the effect of fumonisin concentrations between 21.9 and 32.7 mg/kg.

## 1. Introduction

Fumonisins are a group of mycotoxins mainly produced by *Fusarium vertcilliodes* and *F. proliferatum*. There are 28 fumonisin homologs, and they are highly polar compounds and soluble in water. Fumonisin B1 (FB1) is the most prevalent and toxic of the fumonisin family, while fumonisin B2 and B3 are less prevalent and are usually associated with FB1 but at lower concentrations. Fumonisin contamination in corn has been an emerging issue in animal feed production [1]. Pigs fed fumonisin-contaminated corn have reduced growth performance, and damage to the liver, lungs [2], kidneys [3], and gastrointestinal structure [4]. According to previous research, less than 25 mg/kg of fumonisins causes no apparent clinical changes, but when pigs were fed more than 50 ppm reduced growth performance and liver damage were observed [5]. High doses (above 100 mg/kg of feed) of fumonisin contamination in swine feed for a short period of time (approximately 3 to 5 days) can cause acute porcine pulmonary edema (PPE), which is often lethal [5]. The U.S. Food and Drug Administration established a guidance level of 20 mg/kg total fumonisins (B1 + B2 + B3) as a maximum in corn used for swine feed and at this level of contamination, corn should not exceed 50% of the diet [6]. The European Commission also established a recommendation for fumonisin (B1 + B2) levels for corn used in animal feed to be below 60 mg/kg and no more than 5 mg/kg in complete swine diets [7].

Fumonisins have a similar structure to sphinganine (SA) and sphingosine (SO), therefore they can act as a competitive inhibitor for ceramide synthase, a crucial enzyme for synthesizing complex sphingolipids from SA (via de novo synthesis) and SO (via sphingolipid turnover) in animals [8]. After fumonisin ingestion and absorption, the toxin accumulates in tissues, such as lung, liver, kidneys, brain, and fat tissue. Liver and kidneys are the preferred organs for fumonisin B1 accumulation [9]. Sphingolipids are important components for cell membrane and lipid-rich structures. They serve as binding sites for extracellular matrix proteins, modulators for growth factor receptors, and precursors for second messengers of growth factor and other cell responses [8]. Disrupted sphingolipid metabolism causes cell damage and apoptosis in organs, such as the liver and kidney [9]. Without being converted to sphingolipids by ceramide synthase, SA and SO accumulate in tissue. Sphinganine accumulates at a greater rate than SO, therefore increased SA to SO ratios can be observed in these tissues with increased fumonisin concentration [10]. Individual sphinganine and sphingosine concentrations vary between individuals, but SA to SO ratios are relatively stable and have been proposed as an indicator for the severity of fumonisin toxicosis [11,12,13]. Generally, serum SA:SO ratios of control pigs without fumonisin intoxication range from undetectable to 1.08 [12]. To our knowledge, there is limited data on the concentration of naturally fumonisin-contaminated corn that affects pig growth performance and serum SA:SO ratio [14,15]. Because co-occurrence of fumonisin with other mycotoxins in nature is common [16], most previous studies have determined the effects of fumonisin on pigs by utilizing purified fumonisin toxin or cultured feed material [17,18,19], but not corn naturally contaminated with fumonisins. Therefore, the objective of this study was to determine the effects of feeding corn naturally contaminated with fumonisins on the growth performance and SA:SO ratio of 9 to 28 kg nursery pigs.

## 2. Results and Discussion

The corn used in this study had high naturally contaminated fumonisin concentrations without detectable levels of other mycotoxins (Table 1). This allowed us to exclude the interactive effects of multiple mycotoxins on growth performance and the serum SA:SO ratio. The dietary fumonisin (B1 + B2) levels were 7.2, 14.7, 21.9, 32.7, and 35.1 mg/kg. The ratios of fumonisin B1 to B2 ranged from 3.4 to 4:1, which were approximately the same as the previously reported 3:1 ratio [20]. Fumonisin B3 was not analyzed due to its much lower toxicity compared to fumonisin B1 and B2 [5]. The diet with 7.2 mg/kg of fumonisins (B1 + B2) was manufactured with corn with the lowest mycotoxin contamination that could be obtained locally in the same crop year, which suggested that this corn still contained about 10 mg/kg of fumonisins (B1 + B2). Corn with 10 mg/kg of fumonisins may not pose an immediate danger to swine but would endanger equines due to their higher sensitivity toward fumonisins [5]. The highest fumonisin diet (35.1 mg/kg of B1 + B2) suggests that the high fumonisin-contaminated corn contained about 50 mg/kg of fumonisins (B1 + B2). This level would cause negative effects on pigs fed common corn-soybean meal diets according to previous researchers [5].

Increasing dietary fumonisin (B1 + B2) concentration from 7.2 to 35.1 mg/kg for 28 days decreased (linear, *p* < 0.001) the overall average daily gain (ADG) and day 28 body weight (BW), overall average daily feed intake (ADFI; linear, *p* = 0.055), and overall gain to feed ratio (G:F; linear, *p* = 0.016; Table 2). Serum SA:SO ratios on day 14 and 28 were linearly increased (*p <* 0.001) as dietary fumonisin (B1 + B2) concentration increased (Table 3). Although testing linear, the greatest reduction in overall growth performance and increase in serum SA:SO ratios were observed in pigs fed 32.7 and 35.1 mg/kg of fumonisins (B1 + B2). There were no apparent external clinical signs of fumonisin intoxication, such as coughing, except for reduced growth performance and increased serum SA:SO ratio. There was no evidence of difference on the percentage of pigs receiving injectable antibiotic treatment or number of pigs removed from the study between treatments (data not shown). Even though mycotoxin-contaminated corn may have reduced oil content [21], the chemical analysis of the diets showed no difference in major nutrient values among treatments (Table 4). Therefore, the reduced growth performance is likely not a function of nutrient value differences between the two corn sources, but is possibly caused by disrupted gastrointestinal function and other negative effects on organs, such as the heart, liver, and kidneys attributed to the increased fumonisins.

**Figure 1:**
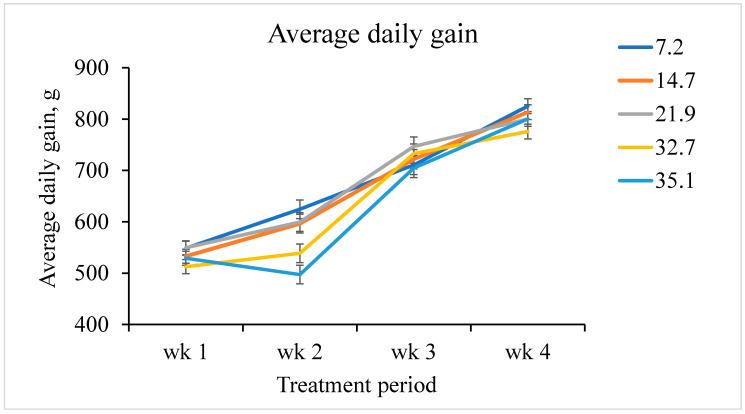
Weekly average daily gain of pigs fed increasing fumonisin concentration. The dietary fumonisin levels were 7.2, 14.7, 21.9, 32.7, and 35.1 mg/kg of fumonisin (B1 + B2). There was a linear fumonisin concentration × week interactive effect (*p <* 0.001). From day 0 to 7, there was no evidence of a linear effect (*p* = 0.182) and a quadratic effect (*p* = 0.785). From day 7 to 14, there was a linear effect (*p <* 0.001) and a tendency of a quadratic effect (*p* = 0.084). From day 14 to 21, there was no evidence of linear effect (*p* = 0.888), but a tendency to a quadratic effect (*p* = 0.077). From day 21 to 28, there was a tendency to a linear effect (*p* = 0.053) and no evidence of a quadratic effect (*p* = 0.615).

**Figure 2:**
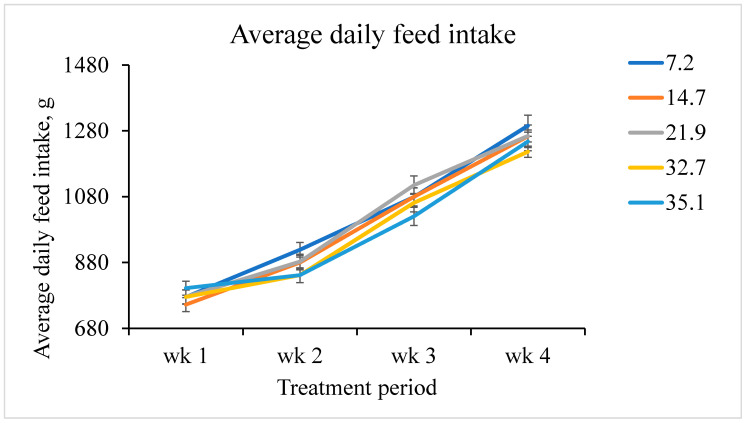
Weekly average daily feed intake of pigs fed increasing fumonisin concentration. The dietary fumonisin levels were 7.2, 14.7, 21.9, 32.7, and 35.1 mg/kg of fumonisin (B1 + B2). There was a linear fumonisin concentration × week interactive effect (*p* = 0.008). From day 0 to 7, there was no evidence of a linear effect (*p* = 0.328) or a quadratic effect (*p* = 0.427). From day 7 to 14, there was a linear effect (*p* = 0.007), but no evidence of a quadratic effect (*p* = 0.892). From day 14 to 21, there was a tendency to a linear effect (*p* = 0.077) and a quadratic effect (*p* = 0.041). From day 21 to 28, there was a linear effect (*p* = 0.030), but no evidence of a quadratic effect (*p* = 0.698).

**Figure 3:**
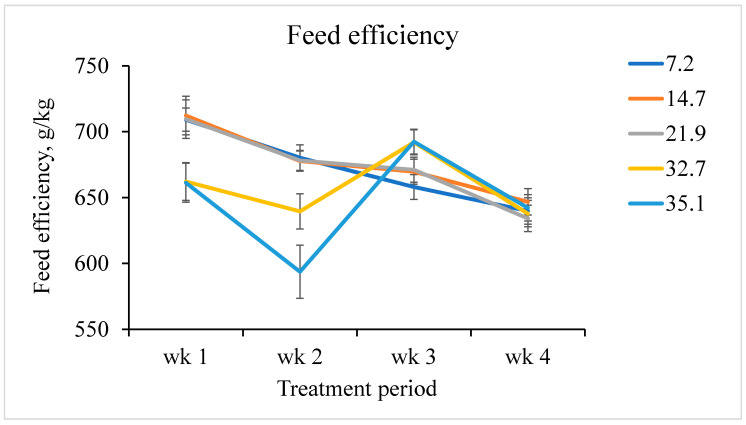
Weekly feed efficiency of pigs fed increasing fumonisin concentration. The dietary fumonisin levels were 7.2, 14.7, 21.9, 32.7, and 35.1 mg/kg of fumonisin (B1 + B2). There was a linear fumonisin concentration × week interactive effect (*p <* 0.001). From day 0 to 7, there was a linear effect (*p <* 0.001) and a tendency to a quadratic effect (*p* = 0.051). From day 7 to 14, there was a linear effect (*p <* 0.001) and a quadratic effect (*p* = 0.004). From day 14 to 21, there was a linear effect (*p* = 0.010), and no evidence of a quadratic effect (*p* = 0.826). From day 21 to 28, there was no linear effect (*p* = 0.877) and no quadratic effect (*p* = 0.864).

The interactive effects of increasing fumonisins on ADG, ADFI, and G:F by week were also assessed. A linear fumonisin concentration × week interaction was observed (*p <* 0.001) for ADG (Figure 1). Increasing fumonisin concentration linearly reduced ADG from day 7 to 14, but not during any other week. A linear fumonisin concentration × week interaction was also observed (*p* = 0.008) for ADFI (Figure 2). Increasing fumonisin concentration linearly reduced ADFI from day 7 to 14 and day 21 to 28, but not from day 0 to 7. A linear fumonisin × week interaction was also observed *(p <* 0.001) for G:F (Figure 3). Increasing fumonisin concentration linearly reduced G:F from day 0 to 7 and day 7 to 14, but not from day 14 to 21 and day 21 to 28. Pigs fed 21.9 mg/kg fumonisins or less had a normal reduction in G:F over the course of the experimental period (Figure 3); however, pigs fed 32.7 or 35.1 mg/kg fumonisins had severe decreases in G:F from day 0 to 7 and day 7 to 14 compared with other treatments. Feed efficiency for these treatments appeared to recover from day 14 to 21 to be similar to pigs fed 21.9 mg/kg fumonisins or less. The weekly performance suggests that ADG and ADFI was less affected by fumonisins during the first week than during the second week. This indicates that, for this range of fumonisin concentration, it took more than a week to cause a significant reduction in growth performance. This suggests that the reduction in growth performance might not be due to the poorer palatability of feed containing high fumonisins, but due to other negative effects caused by fumonisin intoxication. During the last two weeks (day 14 to 28), some recovery of growth performances was observed. This suggests that after two weeks of fumonisin intoxication, pigs fed high fumonisin diets might be able to acclimate to these fumonisin concentrations. However, the mechanism of increasing tolerance is unknown, and the reduction in ADG and subsequent BW loss were not recovered by day 28.

Gbore [22] fed pigs with diets containing cultured fumonisin B1 from 0.2 to 15 mg/kg for 6 weeks and observed similar linear reductions in growth performance (BW, ADG, ADFI, and G:F) and also found delayed sexual maturity in weaned pigs fed high fumonisin levels. A linear reduction in ADG was also observed in 10 kg nursery pigs when fed 0 to 10 mg/kg of purified fumonisin B1 and pigs fed 10 mg/kg fumonisins had an increased organ SA:SO ratio [23]. Nursery pigs fed diets containing 10 mg/kg of cultured fumonisin B1 for 4 weeks showed a reduction in BW, feed intake, G:F, and an increase in lung and liver lesions [24]. In another study, pigs exposed to 25 to 30 mg/kg of cultured fumonisin B1 for 9 days also had a reduction in G:F [25], which matched our finding that G:F was negatively affected in the first week while ADG and ADFI were not. However, Zomborszky et al. [11] used diets for weaned pigs that contained 0 to 40 mg/kg of cultured fumonisin B1 for 4 weeks or 0 to 10 mg/kg of cultured fumonisin B1 for 8 weeks [2] and observed no evidence of differences in growth performance, even though there were increasing signs of pathological changes in lungs of some pigs fed more than 10 mg/kg of fumonisin B1.

In our study, all selected pigs had serum SA:SO ratios below 1:1 on day 0. We observed that increasing fumonisin concentration increased (linear, *p* < 0.001) serum SA:SO ratio from 0.47 to 1.40 on day 14 and 0.55 to 1.58 on day 28. By correlating our growth performance results with serum SA:SO ratios, there was a threshold of approximately 20 to 30 mg/kg of dietary fumonisins (B1 + B2) that resulted in the greatest reduction in growth performance, which corresponded with serum SA:SO ratios over 1:1, the ratio value that is generally classified as fumonisin intoxication. Dilkin et al. [13] observed that the plasma SA:SO ratios of 25 kg pigs were increased 12-fold to 1.11:1 at 6 h after a single dose of fumonisin B1 (5 mg/kg of BW, equivalent to 83 mg/kg of feed). This indicates that plasma SA:SO is a quick and sensitive biomarker for fumonisin toxicosis. Schertz et al. [19] fed 34 kg pigs a single dose of cultured fumonisins (B1 + B2) at 2.5 mg/kg BW and observed significantly elevated serum SA:SO ratios at 24 h post dosing and continued to increase at 120 h post dosing (end of the trial) with no evidence of differences in other blood criteria or lung lesions. Zomborszky et al. [11] fed 0, 10, 20, and 40 mg/kg of cultured fumonisin B1 to 10 kg pigs and observed increasing SA:SO ratios as fumonisins increased on day 3 of the trial, and the degree of difference between treatments increased until the last day (day 8) of the trial. Riley et al. [14] also observed that serum SA:SO increased significantly when pigs were fed fumonisins at 23 mg/kg or greater of purified fumonisins (B1 + B2) after 5 days, which matched our serum SA:SO results. Even though Riley et al. [14] observed elevated serum SA:SO ratios, pigs did not develop lesions in liver, lung, and kidney when fed 23 mg/kg of fumonisins (B1 + B2). Meanwhile liver lesions were observed when pigs were fed 39 mg/kg or greater of fumonisins (B1 + B2) and lung lesions were observed when pigs were fed 175 mg/kg of fumonisins (B1 + B2). These results indicate that the higher the fumonisin level, the faster the SA:SO ratio increases. Therefore, serum SA:SO ratio has the potential to be used as a reliable and quick biomarker for fumonisin’s negative effect on pig growth performance without euthanizing the animals for autopsy.

In conclusion, our results for the effects of feeding pigs with naturally fumonisin-contaminated corn on growth performance and serum SA:SO ratio match previous studies where pigs fed less than 25 ppm of purified or cultured fumonisins had minimal changes in clinical chemistry [5]. While the response to increasing fumonisin in the current study was linear, there were minimal effects on growth performance in pigs fed up to 21.9 mg/kg. The greatest decrease in average daily gain was observed in pigs fed diets that contained more than 32.7 mg/kg of fumonisin (B1 + B2). Diets containing more than 21.9 mg/kg should be evaluated with caution as further research is warranted to determine the fumonisin concentration between 21.9 and 32.7 mg/kg where the negative effects on pig performance and serum SA:SO ratio are most evident. Moreover, pigs ingesting fumonisins below 21.9 ppm for more than 28 days still need further evaluation to determine the effect of long term fumonisin intoxication.

## 3. Materials and Methods

### 3.1. Ethics Statement

The Kansas State University Institutional Animal Care and Use Committee approved the protocol used in this experiment (Approval no. 4036.25 approved 3/6/2019). This experiment was conducted at the Kansas State University Swine Teaching and Research Center in Manhattan, KS. Each pen (1.2 × 1.2 m) provided 0.28 m^2^ per pig and was equipped with a 4-hole, dry self-feeder, and a nipple waterer to provide ad libitum access to feed and water.

### 3.2. Animal and Experimental Design

A total of 350 pigs (241 × 600; DNA, Columbus, NE; initially 8.9 kg) were used in a 28-day growth trial. Pigs were weaned at approximately 21 days of age and placed in pens of 5 pigs each based on initial weight and gender. A common phase 1 pelleted diet was fed for 7 days and a common phase 2 mash diet was fed for another 14 days. At day 21 after weaning, which was considered day 0 of the trial, pens of pigs were randomly allotted to treatment in a randomized complete block design with weight as the blocking factor. There were 14 replicate pens per treatment. Pen weights and feed disappearance were measured weekly to determine ADG, ADFI, and G:F. 

All diets were manufactured at the Kansas State University O.H. Kruse Feed Technology Innovation Center in Manhattan, KS. Two diets were formulated using low fumonisin corn (10 mg/kg of fumonisin B1 + B2; control) or fumonisin-contaminated corn (approximately 50 mg/kg fumonisin B1 + B2; Table 5). For both corn sources, Aflatoxin B1, aflatoxin B2, aflatoxin G1, aflatoxin G2, T-2 toxin, ochratoxin, and sterigmatocystin were below detectable concentration (<20 ug/kg); HT-2 toxin and vomitoxin were below detectable concentration (<200 ug/kg); and zearalenone was below detectable concentration (<100 ug/kg). These two diets were blended at the feed mill to produce three additional diets with intermediate fumonisin concentrations. Consequently, five dietary treatments were manufactured with final diets containing 7.2, 14.7, 21.9, 32.7, and 35.1 mg/kg of analyzed fumonisin (B1 + B2). All diets were formulated to contain 1.30% standardized ileal digestible lysine and methionine or exceeded the NRC [26] nutrient requirement estimates.

Representative diet samples were obtained from every fifth bag of feed manufactured. Samples were analyzed for dry matter (method 935.29) [27], crude protein (method 990.03) [27], Ca (method 6.3) [28], *p* (method 6.3) [28], neutral detergent fiber [29], and ether extract [30] (Laboratories Inc., Kearney, NE, USA, Table 4).

### 3.3. Mycotoxin Analysis

Representative complete diet samples were sent to North Dakota State University Veterinary Diagnostic Laboratory (Fargo, ND) for mycotoxin analysis. The multiple mycotoxin assay is based on an Agilent Technologies method for mycotoxin in maize using ultra high-pressure liquid chromatography and tandem mass spectrometric detection (UHPLC-MS/MS) with modifications [31]. It was adapted to quantitate the following mycotoxins: aflatoxins B1, B2, G1, G2, fumonisins (FB1, FB2), ochratoxin, deoxynivalenol, zearalenone, T-2 toxin, HT-2, citrinin (screen not quantitative) and sterigmatocystin. Matrix effects were compensated for by using 13-carbon-labeled (13C) mycotoxins as an internal standard for each of the 13 target compounds. The standards and 13C-labeled standards were purchased from Romer Labs (Biopure™, Getzersdorf, Austria). The limits of quantitation (LOQ) for this UHPLC/MS/MS assay at parts per billion (ppb) levels vary with the mycotoxin but are consistent across all matrices, are below concentrations listed in the US Food and Drug Administration (FDA) guidelines for mycotoxins in animal feeds, and are at practical concentrations for mycotoxins detected in animal feeds in the US. Twenty-five grams of ground and homogenized feed sample was extracted in acetronitrile (LC-MS grade)/nanopure water (50/50, *v*/*v*) containing 0.1% formic acid. Samples were shaken for 60 min, diluted with extracting solution, centrifuged, and a 13-C internal standard was added to final extract. Analysis was carried out on the Agilent 6460 Triple Quad LC/MS (Agilent Technologies Inc., Santa Clara, CA, USA) with positive electrospray ionization in Dynamic MRM (multiple reaction monitoring) mode using two major transitions per target compound and one transition for the 13C-labeled internal standards.

### 3.4. Serum SA and SO Analysis

Serum samples were collected on day 0, 14, and 28 for the determination of serum SA:SO ratio. Blood samples were collected from two pigs per treatment and analyzed as a baseline concentration for all treatments on day 0. Blood samples were collected from nine pigs per treatment on day 14 and 28. For each selected pen, the median weight pig was selected. The same pig per pen was bled for the day 14 and 28 collections. Whole blood samples were allowed to clot for 30 min, centrifuged at 1500× *g* 1500× *g* for 15 min. and the resulting serum supernatants were transferred to polypropylene tubes and stored at −80 °C. Serum samples were sent to the University of Missouri Veterinary Medical Diagnostic Laboratory (Columbia, MO) and analyzed for sphinganine/sphingosine by HPLC with fluorescence detection utilizing a modification of the method of Hsiao et al. [12] and Riley et al. [32]. Briefly, 0.5 mL of serum sample was transferred to 15 mL polypropylene tubes and 2.0 mL of methanolic 0.125M KOH and 500 μL of chloroform was added. The samples were vortexed and incubated in a water bath at 37 °C for a minimum of 30 min. Sphinganine and sphingosine were extracted by adding 2.0 mL chloroform, 2.0 mL alkaline water (200 μL of 2 N NH_4_OH in 100 mL DD water), and 500 µL of 2N ammonium hydroxide. The samples were vortexed, centrifuged at 224× *g* for 10 min and 2 mL of the lower chloroform layer was transferred to polypropylene vials containing 4 mL alkaline water. The vials were vortexed, centrifuged for 10 min at 224× *g* and the upper phases were discarded, and the lower chloroform layers were evaporated to dryness. The residues were reconstituted in 600 μL of 80% methanol and transferred to autosampler vials. OPA (o-phthaldialdehyde) reagent (300 μL) was added and the derivatized samples were analyzed along with appropriate standards by HPLC. The HPLC system consisted of a Hitachi Model L-7100 pump, Hitachi Model L-7485 fluorescence detector (ex-230 nm; em-430 nm), Hitachi Model L-7200 autosampler with Hitachi D-7000 data acquisition interface and ConcertChrom software. The HPLC column was a 250 × 4.6 Synergi 4 um Polar RP (Phenomenex) with a C18 SecurityGuard precolumn (Phenomenex) and a mobile phase of methanol: 0.005 M K2HPO4 buffer (pH7.0) (850:150) at a flow rate of 0.8 mL/min.

### 3.5. Statistical Analysis

Data were analyzed as a randomized complete block design with block as a random effect and pen as the experimental unit. For every response, two analytical models were constructed by using homogenous variance and heterogenous variance models weighted by treatment. After comparing the two models, one was selected based on the ANOVA test (*p ≤* 0.05) via Bayesian information criterion. Polynomial contrasts were constructed to evaluate the linear and quadratic effects of increasing fumonisins on ADG, ADFI, G:F, BW, and serum SA:SO ratio. Contrast coefficients were adjusted for unequally spaced treatments. Interactive effects of fumonisin level and growth period (week) was tested as repeated measurement. Data were analyzed using the R program [33]. Results were considered significant at *p* ≤ 0.05 and marginally significant at 0.05 < *p* ≤ 0.10.

## Figures and Tables

**Table 1:** Dietary mycotoxin level (as-fed basis) ^1,2,3^.

Item, mg/kg	Analyzed Fumonisin Concentration, mg/kg				
	7.2	14.7	21.9	32.7	35.1
Fumonisin B1	5.68	11.71	17.35	25.21	27.46
Fumonisin B2	1.49	2.96	4.51	7.46	7.54

^1^ A representative sample of each diet was collected from every fifth bag of feed manufactured for each treatment. ^2^ Diet mycotoxin concentration was analyzed at North Dakota State University Veterinary Diagnostic Laboratory (Fargo, ND) by LC/MS/MS assay. All mycotoxins except fumonisin were below detectable level. ^3^ Aflatoxin B1, aflatoxin B2, aflatoxin G1, aflatoxin G2, T-2 Toxin, ochratoxin, and sterigmatocystin were below detectable concentration (<20 ug/kg); HT-2 toxin and vomitoxin were below detectable concentration (<200 ug/kg); and zearalenone was below detectable concentration (<100 ug/kg).

**Table 2:** Effect of fumonisin concentration on 9 to 28 kg nursery pig growth performance ^1,2^.

	Analyzed Fumonisin Concentration, mg/kg						Probability, *p*	
Item	7.2	14.7	21.9	32.7	35.1	SEM	Linear	Quadratic
BW, kg								
d 0	8.9	8.9	8.9	8.9	8.9	<0.21 ^3^	0.598	0.724
d 28	28.1	27.7	27.8	26.8	26.6	0.42	<0.001	0.410
d 0 to 28								
ADG, g	677	666	674	640	633	10.4	0.001	0.184
ADFI, g	1016	993	1010	974	978	18.6	0.055	0.774
G:F, g/kg	667	672	668	658	648	6.4	0.016	0.114

^1^ A total of 350 pigs (241 × 600; DNA, Columbus, NE; initially 8.9 kg) were used in a 28-day experiment with 5 pigs per pen and 14 pens per treatment. ^2^ ADG = average daily gain. ADFI = average daily feed intake. G:F = feed efficiency. ^3^ Heterogenous SEM (standard error of the mean): 7.2 mg/kg (0.19), 14.7 mg/kg (0.19), 21.9 mg/kg (0.18), 32.7 mg/kg (0.21), and 35.1 mg/kg (0.19).

**Table 3:** Effect of fumonisin concentration on the serum sphinganine (SA) to sphingosine (SO) ratio ^1,2,3^.

	Analyzed Fumonisin Concentration, mg/kg						Probability, *p*	
Item	7.2	14.7	21.9	32.7	35.1	SEM	Linear	Quadratic
SA:SO								
d 14	0.47	0.84	1.00	1.14	1.40	0.09	<0.001	0.364
d 28	0.55	0.77	0.93	1.42	1.58	<0.15 ^4^	<0.001	0.143

^1^ A total of 350 pigs (241 × 600, DNA, Columbus, NE; initially 8.9 kg) were used in a 28-day experiment with 5 pigs per pen and 14 pens per treatment. ^2^ Two pigs per treatment were sampled and analyzed as baseline for all treatments on d 0 (SA/SO = 0.22); 9 pigs per treatment were sampled and analyzed on d 14 and 28. ^3^ Serum SA to SO ratio was analyzed at the University of Missouri Veterinary Medical Diagnostic Laboratory (Columbia, MO) by HPLC. ^4^ Heterogenous variance: 7.2 mg/kg (0.03), 14.7 mg/kg (0.07), 21.9 mg/kg (0.08), 32.7 mg/kg (0.07), and 35.1 mg/kg (0.15).

**Table 4:** Chemical analysis of diets (as-fed basis) ^1,2^.

	Analyzed Fumonisin Concentration, mg/kg				
Item, %	7.2	14.7	21.9	32.7	35.1
Dry matter	87.54	87.56	87.72	87.92	88.14
Crude protein	19.3	19.3	19.8	19.7	19.8
Ca	0.73	0.65	0.57	0.67	0.63
P	0.51	0.49	0.50	0.51	0.53
Neutral detergent fiber	6.5	5.6	6.6	6.8	6.3
Ether extract	5.1	4.9	4.8	5.0	5.1

^1^ A representative sample of each diet was collected from every fifth bag of feed manufactured for each treatment, homogenized, and submitted for proximate analysis (Ward Laboratories, Inc., Kearney, NE). ^2^ Diet mycotoxin concentration was analyzed at North Dakota State University Veterinary Diagnostic Laboratory (Fargo, ND) by LC/MS/MS assay. All mycotoxins except fumonisin were below detectable level.

**Table 5:** Diet composition (as-fed basis) ^1,2^.

	Analyzed Fumonisin Concentration, mg/kg	
Item	7.2	35.1
Ingredients, %		
Corn, 10 mg/kg fumonisin B1 + B2	64.70	--
Corn, 50 mg/kg fumonisin B1 + B2 ^3^	--	64.70
Soybean meal	28.00	28.00
Soybean oil	3.00	3.00
Monocalcium phosphate	0.85	0.85
Calcium carbonate	0.75	0.75
Sodium chloride	0.60	0.60
L-Lysine HCl	0.55	0.55
DL-Methionine	0.21	0.21
L-Threonine	0.23	0.23
L-Tryptophan	0.06	0.06
L-Valine	0.16	0.16
Vitamin premix ^4^	0.25	0.25
Trace mineral premix ^4^	0.15	0.15
Phytase ^5^	0.08	0.08
Total	100	100
Calculated analysis Standardized ileal digestible amino acids, %		
Lysine	1.30	1.30
Isoleucine:lysine ^6^	53	53
Leucine:lysine	111	111
Methionine:lysine	36	36
Methionine and cysteine:lysine	56	56
Threonine:lysine	63	63
Tryptophan:lysine	20.0	20.0
Valine:lysine	69	69
Histidine:lysine	35	35
Net energy, kcal/kg	2536	2536
Crude protein, %	19.8	19.8
Calcium, %	0.61	0.61
STTD P ^7^, %	0.44	0.44

^1^ Diet mycotoxin concentration was analyzed at North Dakota State University Veterinary Diagnostic Laboratory (Fargo, ND) by LC/MS/MS assay. All mycotoxins except fumonisin were below detectable level. ^2^ The 7.2 and 35.1 mg/kg fumonisin treatments were blended to manufacture diets with intermediate fumonisin level (14.7, 21.9, and 32.7 mg/kg of fumonisin). ^3^ Approximately 50 mg/kg of fumonisin B1 + B2. ^4^ Provided per kg complete feed: 4133 IU vitamin A; 1653 IU vitamin D3; 44 IU vitamin E; 3 mg vitamin K; 0.03 mg vitamin B12; 50 mg niacin; 28 mg d-pantothenic acid; 8 mg riboflavin; 0.30 mg selenium; 16 mg Cu from copper sulfate; 110 mg Fe from iron sulfate; 110 mg Zn from zinc sulfate; 33 mg Mn from manganese sulfate; 0.3 mg I from Ca iodate. ^5^ Ronozyme HiPhos 2700 (DSM Nutritional Products, Basel, Switzerland) provided 2027 FTU per kg of feed and an expected STTD *p* release of 0.12%. ^6^ Values represent the amount of the individual amino acid relative to the concentration of Lysine. ^7^ STTDP = standardized total tract digestible phosphorus.
